# Chinese Younger Parents' Quality of Life During the COVID-19 Pandemic: Do Job Changes and Family Conflicts Matter?

**DOI:** 10.3389/fpubh.2021.758242

**Published:** 2022-01-21

**Authors:** Xiaohan Liu, Yashuang Bai, Ning Huang, Farooq Ahmed, Muhammad Shahid, Jing Guo

**Affiliations:** ^1^Department of Health Policy and Management, School of Public Health, Peking University, Beijing, China; ^2^Department of Anthropology, Quaid-I-Azam University, Islamabad, Pakistan; ^3^Department of Anthropology, University of Washington, Seattle, WA, United States; ^4^School of Insurance and Economics, University of International Business and Economics (UIBE), Beijing, China

**Keywords:** quality of life, job changes, family conflict, Chinese parents, COVID-19

## Abstract

The quality of life (QoL) might have been decreased owing to social disruptions in daily life and basic functioning after the coronavirus disease (COVID-19) pandemic. This work aims to examine the relationship between job changes, family conflicts, and QoL among parents during COVID-19 in China. We recruited 1,209 adults through an online cross-sectional survey in China during the COVID-19 lockdown from April 21 to April 28, 2020. Convenient and cluster sampling methods were used to recruit parents. The global health items in the Patient-Reported Outcomes Measurement Information System (PROMIS) were used as a measurement for QoL. Data were mainly analyzed by multiple linear regression with SPSS. Both marital conflict (β = −0.243, *p* < 0.001) and parent–child conflict (β = −0.119, *p* = 0.001) were negatively associated with the QoL among parents during the lockdown. Job changes moderated the relationship between marital conflict and QoL (β = −0.256, *p* = 0.022). In addition, the interaction effects of job changes and family conflict on QoL were significant only among fathers and one-child families. This study indicated that family conflict was a crucial factor correlated with QoL among young parents in the backdrop of the COVID-19 lockdown. Job changes could interact with marital conflict and parent–child conflict on the quality of life.

## Introduction

Coronavirus disease 19 (COVID-19), which emerged in Wuhan, China, has reached the level of a pandemic, attracting enormous concern from around the world (1). Several countries took drastic mitigation measures, including community-wide lockdowns, home quarantines, closure of schools, working-from-home, and social distancing to protect the population (2). However, these swift actions have created a host of new challenges that have brought profound changes and affected the normal routines of daily work and lifestyles for people, such as restricting outdoor activities or increasing family conflicts, reducing income, high rates of unemployment (3), and consequently worsening the quality of life (QoL) (4).

Quality of life is a multidimensional concept that usually includes individuals' physical health, psychological state, and level of independence (5). Widespread outbreaks of COVID-19 may adversely affect the multidimensional QoL, especially in parents who have children below 10 years old. Emerging research has shown that parents of young children are more affected by the COVID-19 pandemic (6). Since young children need more supervision and care, the pandemic may bring a unique challenge to parents of young children (7). Specifically, parents with young children may have more childcare responsibilities at home due to the reduced social network and the swift closure of schools and childcare centers (7). In addition, they had to balance remote working with home-schooling their children and confront more work–family conflict (8). Thus, in this difficult time, more attention is required to the younger parents' QoL.

### Job Changes and QoL

Job changes are significant life stressors, and losing a job takes several years to recover psychologically (9). According to the family stress model (10), economic instability (including unemployment, debt, and receipt of income transfers) places considerable strain on parents. The COVID-19 pandemic has forced parents to quickly transition to a new way of work (11). These transitions present all kinds of stressors, which might have repercussions on parents' capacities to be psychologically flexible, thus putting their QoL at risk (11).

### Family Conflict and QoL

Active opposition of various forms, including verbal, physical, sexual, financial, or psychological, among family members is often referred to as family conflict (12). According to adult attachment theory, high-quality close relationships, including marital relationships and parent–child relationships, have been consistently linked to better health, while conflicting relationships may influence parents' physiological responses to stress, risky health behaviors, susceptibility to physical illness, and poorer disease outcomes (13). When parents were asked to stay at home during the pandemic, they faced problems getting along with their family members and dealing with conflicts with their partner and children. There is evidence that a close relationship between family members could affect parents' QoL, and parents' health status can further affect children's development and well-being, creating a vicious circle in such a difficult period (14, 15).

### Conceptual Framework

Theories and empirical results based on role stress theory indicate that the combination of family and employment often creates more demands than one can handle, leading to role overload (16). Consequently, both job changes and family conflict can bring more stress and psychological problems for parents of young children.

The interaction effect of job changes and family conflicts on QoL may be different for mothers and fathers. COVID-19 has indeed changed parents' experiences with employment outside the household and the division of labor at home (17). Compared with fathers, mothers have reduced their working hours more and spent more time on childcare duties (18). Moreover, school and daycare closures increased caregiving responsibilities. Arguably, these changes increased the burden on women more than on men (19). Therefore, this work anticipates that mothers have experienced more job changes, perceived more family conflicts, and low QoL than fathers.

Additionally, the interaction role between job changes and family conflict on QoL may differentiate across the number of children. The traditional belief, “more children, more happiness,” is still entrenched in China (20). Evidence has shown that parents can obtain more emotional support from their children in a family with multiple children (21), and parents with more children perceive a higher level of life satisfaction. In addition, parents tend to rear more children if they can easily integrate work and family roles to reduce the double burden (22). Thus, the association between family conflict and QoL in a multichild family may not be enhanced by job changes.

### Aims and Hypothesis

The objective of this work was to examine the relationship between job changes, family conflict, and QoL among parents with a child aged 0–10, to assess the interaction effects between job changes and family conflict, and to explore the difference in gender and family size among them. We hypothesized that either job changes or family conflicts correlated with parents' poor QoL. We further hypothesized that pandemic-related job changes enhanced the relationship between family conflict and poor QoL. Moreover, we assumed that the abovementioned moderating effects varied across genders and families with different numbers of children.

## Methods

### Study Design and Population

This study was conducted based on an online survey (a web-based platform, https://www.wjx.cn/app/survey.aspx) from April 21 to April 28, 2020, during the COVID-19 lockdown in China. To nationally reflect the mental health status of parents affected by the pandemic and enlarge the sample size, Hubei province, the area most affected by COVID-19, and its neighboring province Henan and non-adjacent province Guangdong were selected for sample investigation.

We used various sampling techniques to reduce bias in situations where there were large populations in China. Random sampling is practically impossible because families have difficulty reaching during the pandemic. The advantages of convenience sampling approaches, such as collecting data quickly, were suitable for data collection during pandemics with school closures. In addition, we used cluster sampling because this method is usually adopted to recruit subjects in the school. Convenience sampling was used to select primary schools. The cluster sampling method was used to choose participants from selected schools. The selected schools were stratified by grade. Then, all classes were selected from each grade of the selected school, and all the students in these classes joined the survey. The head teachers helped us distribute the questionnaires to the subjects online.

Parents aged 18 years and above who had a child or children aged between 0 and 10 years old were invited to participate. The rationale for restricting the sample to parents with at least one 10-year-old or younger child was that younger children necessitate more direct supervision; therefore, it was expected that the COVID-19 pandemic may pose more specific challenges to them, such as more work–family conflict. These parents were instructed about how to answer the questionnaires by online guidance and to complete the questionnaires independently. In total, 1,286 parents participated in the survey. As 77 parents were excluded because they had no job, the final number of participants became 1,209. Each question of the online questionnaire was required to be answered; thus, no missing data were reported in our study. The study protocol was approved by the Institutional Review Board of Peking University, Beijing, China. All participants gave electronic consent after being informed of the aims of the survey and their rights to refuse to participate.

### Measures

Quality of life was assessed with the global health items in the patient-reported outcomes measurement information system (PROMIS) (23). The checklist includes 6 items, which were answered on a five-point scale, Excellent(1)–Poor(5). See the attachment for details. The total scores were calculated by the sum of all items, with a higher score indicative of better QoL. Cronbach's alpha of QoL in this study was 0.916.

Job changes were assessed with a subset of items from the questionnaire of the COVID-19 and perinatal experiences study (COPE Study, https://osf.io/uqhcv/). The scale contained 15 questions about the changes in jobs related to the COVID-19 pandemic, such as telecommuting, reduced working hours, extended working hours, pay cuts, increased responsibility, increased supervision and reporting, and declining job security. For each question, 0 represented “No,” while 1 represented “Yes.” All items were summed to obtain a total score for job changes. A higher score represented more job changes. The internal consistency of job changes was 0.881.

Family conflict was measured with a 6-item self-made questionnaire about specific conflict behaviors, including marital conflict and parent–child conflict. Among them, three questions examined marital conflict, and three other items examined parent–child conflict. Each item was rated on a five-point scale ranging from never (1 score) to always (5 scores). For details, please page to attachment. Higher scores indicated more frequent marital and parent–child conflict. The Cronbach's α were 0.696 and 0.726, respectively. The questionnaire items were vetted by some sociological professors, and they agreed that there were no items that were culturally insensitive.

Confounding variables included demographic and socioeconomic characteristics as follows: exposure to COVID-19 (yes, no), gender (male, female), province (Hubei, Henan, Guangdong, else), number of children (1, 2, 3 or more), education (junior school and below, high school, college, undergraduate, and above), marital status (married, else), occupation (stable, unstable), and annual income ( ≤ 50,000, 50,000–1,00,000, ≥1,00,000). In the study, this term (stable job) refers to work that is continuous and safe employment, and there are no sudden layoffs or labor strife. By unstable jobs we mean work that is uncertain, insecure, irregular, and in which employees bear the risks of work and receive limited social benefits, relatively low income, and statutory protections (i.e., In China, employees work in state-owned enterprises, and public institutions are regarded as having a stable job. Unstable job employees refer to freelancer and gig workers). All covariates were selected according to previous related studies (24, 25).

### Statistical Analyses

Data in this study were analyzed with SPSS version 22.0. Descriptive statistics were calculated to describe the demographic characteristics of the subjects. Since QoL as the dependent variable is continuous, multiple linear regression analysis was performed, and each model controlled the same confounding variables, including exposure, gender, province, number of children, education, marital status, occupation, and annual income.

The main analyses consisted of multiple regressions on QoL in two steps. In model 0, each of the predictors was included separately to estimate their “raw” contribution to the prediction of QoL, and in model 1, the three predictors of job changes, marital conflict, and parent–child conflict were introduced to examine which component would be the most powerful predictor.

In the next multivariable linear regression analysis, job changes, marital conflict, and parent–child conflict and their interaction terms (job changes^*^marital conflict and job changes^*^parent–child conflict) were all standardized and entered to estimate the moderating effects of job changes on the relationships between marital conflict, parent–child conflict, and QoL.

In addition, the whole sample was divided into 2 groups by gender to examine gender differences in the moderating effects of job changes on the relationships between marital conflict, parent–child conflict, and QoL. In the final linear regression models, the whole sample was divided into 3 groups by the number of children in a family.

## Results

The study included 1,209 parents (median age 36 years [SD = 5.16]) in China, 317 (26%) of whom were fathers and 892 (74%) of whom were mothers. A total of 21% of participants were from Hubei. Around 47.2% of the parents reported having one child and 18.7% reported that someone in their family, neighborhood, or friends had suffered or were suffering from COVID-19. Around 6.9% parents did not experience any job changes. Many of parents experienced family conflict (97.7%). The average number of job changes was 17.85, and the mean QoL score was 23.85 (SD = 3.60) (Table 1).

**Table 1:** Descriptive data on social-demographics (*N* = 1,209).

		**Total**		**Father**		**Mother**		***P***
		***N***	**%**	***N***	**%**	***N***	**%**	
Exposure	No	983	81.3	253	79.8	730	81.8	0.200
	Yes	226	18.7	64	20.2	162	18.2	
Parents	Father	317	26.2					0.142
	Mother	892	73.8					
Province	Hubei	256	21.2	71	22.4	185	20.7	0.089
	Henan	182	15.1	54	17.0	128	14.3	
	Guangdong	510	42.2	110	34.7	400	44.8	
	Else	261	21.6	82	25.9	179	20.1	
Number of children	1	571	47.2	163	51.4	408	45.7	0.477
	2	569	47.1	140	44.2	429	48.1	
	3+	69	5.7	14	4.4	55	6.2	
Education	Junior school and below	329	27.2	89	28.1	240	26.9	**0.002**
	High school	222	18.4	43	13.6	179	20.1	
	College	444	36.7	119	37.5	325	36.4	
	Undergraduate and above	214	17.7	66	20.8	148	16.6	
Marital status	Married	1151	95.2	304	95.9	847	95.0	0.226
	Else	58	4.8	13	4.1	45	5.0	
Occupation	Stable job	673	55.7	215	67.8	458	51.3	**0.050**
	Unstable job	536	44.3	102	32.2	434	48.7	
Annual income	≤ 50,000	347	28.7	69	21.8	278	31.2	0.368
	50,000–100,000	376	31.1	99	31.2	277	31.1	
	≥100,000	486	40.2	149	47.0	337	37.8	
		**Mean**	**SD**	**Mean**	**SD**	**Mean**	**SD**	
Parents' age		36.121	5.162	36.852	5.427	35.861	5.042	**0.001**
First child's age		8.393	4.150	8.500	4.436	8.355	4.045	0.119

*Bold values are statistically significant at p <0.05*.

Table 2 shows the results of the multivariable linear regression analysis. In model 0, increased job changes, marital conflict, and parent–child conflict were associated with lower QoL. In model 1, marital conflict explained the largest variance in QoL. More marital conflict was associated with lower QoL (β = −0.243). Additionally, parent–child conflict was inversely correlated with QoL (β = −0.441, *P* = 0.001). However, no significant changes in QoL scores were observed in association with job changes.

**Table 2:** The relationship between job changes, family conflicts, and quality of life.

	**Model 0**				**Model 1**			
	***B***	**SE**	**Beta**	***P***	***B***	**SE**	**Beta**	***P***
**Parents (ref: father)**
Mother	−0.370	0.244	−0.045	0.130	−0.039	0.233	−0.005	0.866
**Number of children(ref: 3+)**
1	0.319	0.486	0.044	0.512	0.332	0.464	0.046	0.475
2	0.006	0.470	0.001	0.989	−0.005	0.446	−0.001	0.990
Job changes	−0.258	0.109	−0.071	**0.019**	−0.076	0.105	−0.021	0.473
Marital conflict	−0.899	0.131	−0.241	**<0.001**	−0.905	0.133	−0.243	**<0.001**
Parent–child conflict	−0.464	0.131	−0.125	**<0.001**	−0.441	0.133	−0.119	**0.001**

*B, non-standardized coefficients; SE, standard error; Beta, Standardized coefficients; bold values are statistically significant at p <0.05; Model 0 is crude estimates, and Model 1 is the estimates accounting for all three independent variables (job changes, marital conflict, and parent–child conflict). All models were controlled for exposure, province, age, education, marital status, income, occupation, and first child age*.

**Figure 1:**
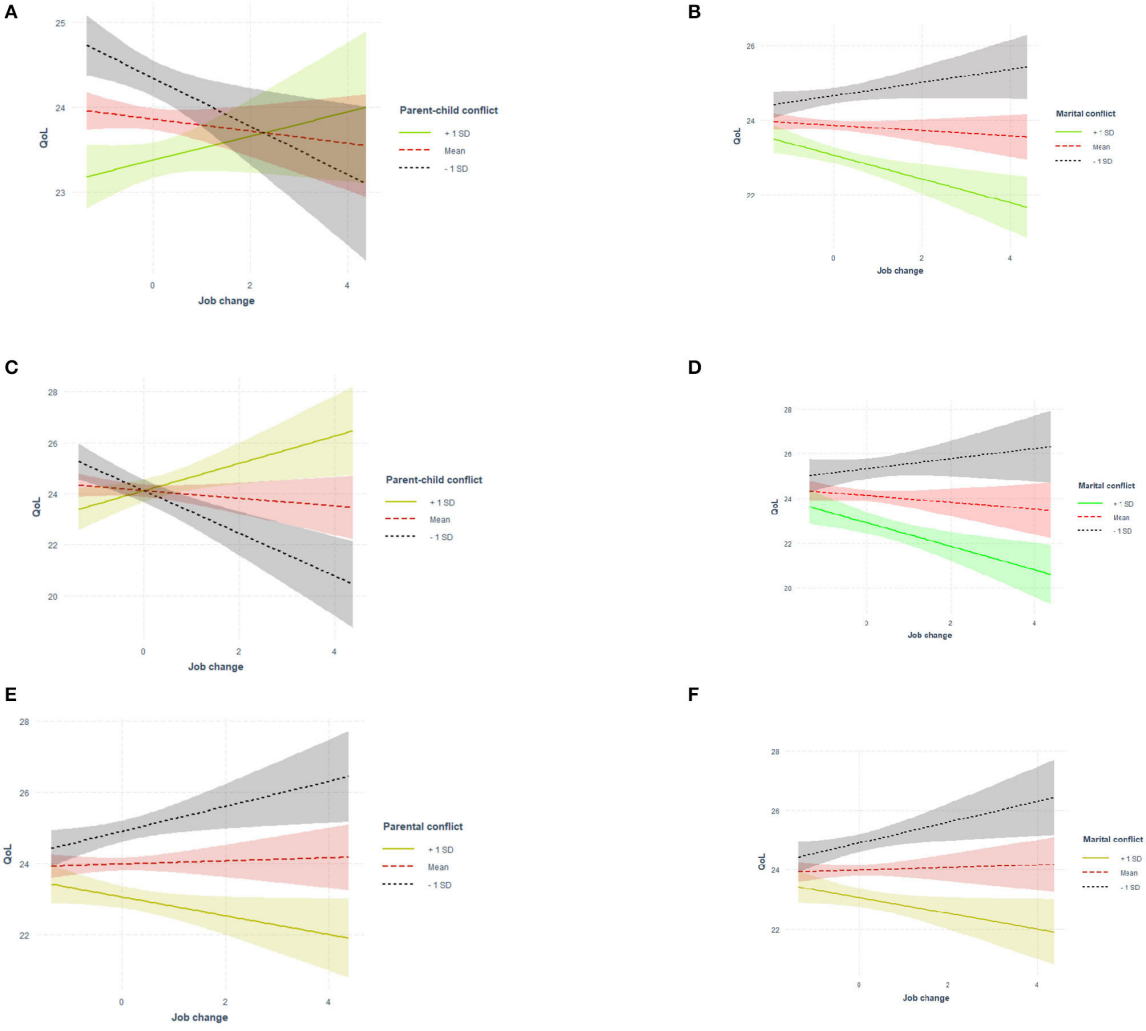
Interaction effect of job change and family conflict. **(A,B)** Whole sample. **(C,D)** Male sample. **(E,F)** One child family.

A significant moderator by family conflict interaction was observed for job changes (Table 3). Parents with more job changes and marital conflict were more likely to receive lower QoL scores (β = −0.256, *p* = 0.022; Figures 1A,B). However, the interaction of job changes and parent–child conflict did not reach significance.

**Table 3:** Analysis of the interaction effects between job changes and family conflicts on quality of life.

	**Model 2**			
	***B***	**SE**	**Beta**	***P***
**Parents (ref: father)**
Mother	−0.067	0.234	−0.008	0.775
**Number of children (ref: 3+)**
1	0.334	0.463	0.046	0.470
2	−0.012	0.446	−0.002	0.979
Job changes	−0.071	0.105	−0.020	0.498
Marital conflict	−0.824	0.137	−0.221	**<0.001**
Parent–child conflict	−0.497	0.135	−0.134	**<0.001**
Job changes* marital conflict	−0.256	0.111	−0.077	**0.022**
Job changes*parent–child conflict	0.219	0.124	0.058	0.077

*B, non-standardized coefficients; SE, standard error; Beta, standardized coefficients; Bold values are statistically significant at p <0.05; All models have been controlled exposure, province, age, education, marital status, income, occupation, first child age*.

Table 4 displays gender differences in the interaction roles between job changes and family conflict. The interaction effects of job changes with marital conflict (β = −0.421, *p* = 0.024) and parent–child conflict (β = 0.747, *p* = 0.002) were only found for fathers, indicating that job changes reinforced the negative effect of marital conflict while alleviating the adverse effect of parent–child conflict on the QoL among fathers (see Figures 1C,D). Table 5 presents the interaction results of job changes with family conflict across families with a different number of children. The moderation effects of job changes with marital conflict and parent–child conflict were found only in the one-child family (β = −0.304, *p* = 0.024 and β = 0.412, *p* = 0.011, respectively). As presented in Figures 1E,F, the negative effect of marital conflict on QoL could be strengthened by job changes among parents with one child, whereas the adverse correlation between parent–child conflict and QoL would be weakened.

**Table 4:** Interaction effects analysis between job changes and family conflicts on quality of life in the gender subgroup.

	**Model 3: father**				**Model 4: mother**			
	***B***	**SE**	**Beta**	***P***	***B***	**SE**	**Beta**	***P***
**Number of children (ref: 3+)**
1	0.889	1.008	0.120	0.378	0.172	0.522	0.024	0.741
2	0.320	0.992	0.043	0.747	−0.002	0.500	0.000	0.997
Job changes	−0.124	0.217	−0.034	0.568	0.003	0.123	0.001	0.980
Marital conflict	−1.354	0.344	−0.326	**<0.001**	−0.672	0.151	−0.186	**<0.001**
Parent–child conflict	0.002	0.315	0.000	0.996	−0.653	0.151	−0.181	<0.001
Job changes * marital conflict	−0.421	0.186	−0.153	**0.024**	0.005	0.151	0.001	0.974
Job changes *parent–child conflict	0.747	0.242	0.188	**0.002**	−0.091	0.152	−0.025	0.550

*B, Non-standardized coefficients; SE, standard error; Beta, Standardized coefficients; Bold values are statistically significant at p <0.05; All models have been controlled exposure, province, age, education, marital status, income, occupation, first child age*.

**Table 5:** Interaction effects analysis between job changes and family conflicts on quality of life in the number of children subgroup.

	**Model 5: one child family**				**Model 6: two children family**				**Model 7: multiple children family (3+)**			
	***B***	**SE**	**Beta**	***P***	***B***	**SE**	**Beta**	***P***	***B***	**SE**	**Beta**	***P***
**Parents (ref: father)**
**Mother**	−0.306	0.317	−0.039	0.334	0.235	0.372	0.028	0.529	1.852	1.310	0.199	0.164
Changes in job	0.075	0.162	0.020	0.643	−0.073	0.156	−0.020	0.640	−0.331	0.453	−0.108	0.469
Marital conflict	−0.857	0.183	−0.254	**<0.001**	−0.738	0.219	−0.180	**0.001**	−1.001	1.162	−0.219	0.393
Parent–child conflict	−0.439	0.189	−0.121	**0.021**	−0.655	0.207	−0.171	**0.002**	−0.411	0.916	−0.116	0.655
Job changes* marital conflict	−0.304	0.135	−0.106	**0.024**	−0.250	0.225	−0.063	0.266	1.230	0.902	0.335	0.179
Job changes*parent–child conflict	0.412	0.161	0.115	**0.011**	−0.017	0.228	−0.004	0.940	−0.395	0.639	−0.127	0.539

*B, Non-standardized coefficients; SE, standard error; Beta, Standardized coefficients; Bold values are statistically significant at p <0.05; All models have been controlled exposure, province, age, education, marital status, income, occupation, first child age*.

## Discussion and Implications

This work focused on the QoL among Chinese parents of young children during the COVID-19 pandemic and examined the relationship between job changes, family conflict, and QoL. Our findings suggested that individuals with more frequent marital conflict and parent–child conflict had worse QoL. We found that job changes significantly enhanced the negative correlation between marital conflict and QoL, but did not significantly buffer the negative relationship between parent–child conflict and QoL among the whole sample. In addition, both of the moderation effects differed across gender and family structure, and they were only significant for fathers and one-child family.

Our results indicated that parents who experienced marital conflict and parent–child conflict reported a lower level of QoL. Marital conflict may decrease marital QoL by increasing negative affect and physiological arousal, according to a social psychophysiological model of marriage (26). In addition, stress process perspectives proposed that chronic strains in the marital role could cause stress, which typically manifests in the form of physical or psychological distress, as indicated by poor QoL (27). Similarly, parent–child conflict might be regarded as a chronic stressor or stressful life event that influences parents' mental health (28). Empirical research has demonstrated that parents in families with a high level of parent–child conflict might more likely suffer depressive symptoms (29), which may reduce parental QoL.

Furthermore, job changes did act as a moderator between family conflict and QoL. On the one hand, job changes accelerated the negative effect of marital conflict on QoL. This could be explained by family stress theory (30). Financial stress that comes with job change, as an uppermost topic of marital disagreement, could cause more emotional distress and then heighten marital conflict (31). Moreover, role theory argues that the role pressures from family and work domains are mutually incompatible in some respects, and job stress would negatively spill over into home life (32), which may cause increased conflicts with spouses. Thus, when facing job changes, individuals who suffer from marital conflict may perceive a lower QoL.

On the other hand, job changes could moderate the negative correlation between parent–child conflict and QoL. Some special features of job changes during COVID-19, such as flexible work time, telework, and working from home, could make these parents have more time to spend with their children at home, thus improving the relationship between parents and children (33). It is also helpful to meet the expectations of parental roles such as caring for children, especially for young Chinese parents who always experience overtime work with less time to interact with family members (34). Therefore, job changes could mitigate the negative effect of parent–child conflict on parents' QoL. Given that the impacts of the COVID-19 pandemic on the economy, work, and home life are manifold and will potentially last for a long time (35), understanding such interactions will be important to provide services for the improvement of QoL among parents.

Of note, the moderating role of job changes was confirmed only for Chinese fathers. Since traditional gender values that “fathers are regarded as the primary breadwinners and mothers as primary caregivers” are still the most far-reaching and prevalent in China (36), work factors would spill over into family more for Chinese men than women, based on gender role theory (37). Specifically, the financial loss coming with job changes during the pandemic may more likely make Chinese husbands as family economic pillars feel stressed than their wives (38), thus enhancing the negative relationship of marital conflict and QoL among fathers. Job changes are more likely to increase positive interactions with children for fathers, such as playing games, which could improve the father–child relationship (39, 40) and thus weaken the association between parent–child conflict and fathers' QoL.

In addition, job changes only moderated the association between family conflict and parents' QoL in the one-child family. This could be explained using resource dilution theory (41); children in the one-child family could receive more attention and resources from parents (42), which means that parents with only children can more easily meet their children's demands using job resources from job changes than parents with multiple children (43). Therefore, job changes could only buffer the adverse effect of parent–child conflict in one-child families.

Moreover, the association between marital conflict and QoL could only be enhanced by job changes in the one-child family. This is because parents with fewer children in collectivistic cultures may have lower marital happiness (44). In addition, previous research examined the quality–quantity trade-off theory based on the relaxation of China's one-child policy (45). This result suggested that Chinese parents with one child might have a strong preference for quality and devote more time, energy, and money to their children's development than other parents (46). Therefore, extended working hours, pay cuts, or unemployment would increase child-rearing pressure on parents with an only child, while the pressure could be mitigated in the multichildren family owing to more support and assistance from children (19).

### Limitation and Implication

Several limitations of this study should be acknowledged. First, the selective bias resulting from convenient sampling methods used in our study might constrain the generalizability of our findings. Second, the proportion of fathers was <30% in our study, which may suggest that we may miss the data from fathers who are quiet busy with their job or other things. Thus, we should be cautious in generalizing our results. Third, our study only included limited job change forms without consulting vocational psychology, which may fail to fully reflect the relationship between job changes and young parents' QoL. Fifth, with the cross-sectional design of the current research, it is difficult to make a causal inference. Longitudinal designs are expected in future studies to help clarify the causal relationships.

Despite these limitations, the findings of this study have significant implications. First, the QoL among young parents experiencing job changes and family conflict should be given adequate attention during COVID-19 confinement, and corresponding proactive and applicable interventions can be proposed. For example, community organizations and social workers should pay more attention to the prevention of family conflict. In addition, fathers and one-child families need to implement psychological interventions (that mitigate marital conflict) and work insurance (that reduce financial pressure).

## Conclusion

This study indicates that family conflict is an important factor related to QoL among younger Chinese parents. Job changes are found to moderate the association between family conflict and QoL. In addition, we find that these interaction effects differ across gender and family structure, and they are only significant for fathers and one-child family. The findings suggest that work insurance programs and professional family support from government and community social workers may be beneficial to improve the QoL of younger parents.

## Data Availability Statement

The raw data supporting the conclusions of this article will be made available by the authors, without undue reservation.

## Ethics Statement

The studies involving human participants were reviewed and approved by Peking University. The patients/participants provided their written informed consent to participate in this study.

## Author Contributions

XL analyzed and interpreted the data and wrote the first draft of the manuscript. YB, FA, MS, and NH commented and revised the manuscript. JG conceptualized the study design, supervised the work, and revised the manuscript. All authors read and approved the final manuscript.

## Funding

This work was supported by the National Social Science Fund of China (Number: 20VYJ042) to JG.

## Conflict of Interest

The authors declare that the research was conducted in the absence of any commercial or financial relationships that could be construed as a potential conflict of interest.

## Publisher's Note

All claims expressed in this article are solely those of the authors and do not necessarily represent those of their affiliated organizations, or those of the publisher, the editors and the reviewers. Any product that may be evaluated in this article, or claim that may be made by its manufacturer, is not guaranteed or endorsed by the publisher.
